# Phosphodiesterase 4-targeted treatments for autoimmune diseases

**DOI:** 10.1186/1741-7015-11-96

**Published:** 2013-04-04

**Authors:** Neal Kumar, Ari M Goldminz, Noori Kim, Alice B Gottlieb

**Affiliations:** 1 Department of Dermatology, Tufts Medical Center, 800 Washington Street #114, Boston, MA, 02111, USA

**Keywords:** Autoimmune, Apremilast, Crohn’s, Dermatitis, PDE4, Psoriasis, SLE

## Abstract

Advancements in phosphodiesterase (PDE)-targeted therapies have shown promise in recent years for treating patients with a variety of autoimmune diseases. This review summarizes the development of PDE4 inhibitors and the associated literature with a focus on treatments for autoimmune diseases. After the initial investigations of the prototypic PDE inhibitor, rolipram, more selective inhibitors targeting the PDE4 isozyme have been developed. With phase II and phase III clinical trials currently underway to evaluate the safety and efficacy of the latest generation of PDE4 inhibitors, namely apremilast, a new class of treatments may be around the corner for patients suffering from chronic, autoimmune diseases.

## Introduction

Our earliest understanding of phosphodiesterase (PDE) inhibitors began with a series of publications by Sutherland and Rall in the 1950s, describing the properties of cyclic adenosine monophosphate (cAMP). Various cellular pathways and inflammatory responses are mediated by cAMP, an essential intracellular second messenger made up of phosphodiester bonds. Evidence showed formation of cAMP was induced by substances such as epinephrine and glucagon, and the suppression of the enzymes hydrolyzing cAMP, including PDEs, by sodium fluoride and caffeine [1,2]. By the 1960s, the role of cyclic nucleotide second messengers, such as cAMP, in cell signaling and homeostasis was established, and regulation of this pathway by PDE inhibitors arose as a field of considerable interest. However, the immunomodulatory properties of cAMP and the anti-inflammatory potential of PDE inhibitors were not demonstrated until the early 1970s [3-5].

Additional research would later demonstrate the expression of the PDE isoenzyme PDE4 almost exclusively within inflammatory cells [6]. Inhibition of PDE4 leads to the reduction of intracellular cAMP levels, and decreases in T cell and monocyte derived cytokine and chemokines including tumor necrosis factor (TNF)α [7-11].

Targeting PDE4 has enormous clinical potential because it targets a central pathogenic process that bypasses complex antigen receptor-specific immunoregulatory mechanisms. Indeed, selective PDE4 inhibitors have generated substantial interest as a treatment for several autoimmune conditions including ankylosing spondylitis, Alzheimer’s disease, psoriasis, psoriatic arthritis, sarcoidosis, systemic lupus erythematosus, inflammatory bowel disease, atopic dermatitis, rheumatoid arthritis, and multiple sclerosis.

### Mechanism of action

PDEs are a family of enzymes responsible for the hydrolysis and subsequent inactivation of cyclic nucleotides, and have been organized into at least 11 families based on sequence homogeneity, inhibitor sensitivity, and biochemical properties [12].

Each enzyme within the PDE4 family specifically targets cAMP for degradation and consists of four subtypes (PDE4A to PDE4D). These enzymes are located within brain and immunocompetent cells such as neutrophils, T lymphocytes, macrophages and eosinophils [13].

PDE4 inhibition results in the accumulation of the intracellular second messenger cAMP, downstream activation of protein kinase A (PKA), and subsequent phosphorylation of the transcription factor cAMP-response element binding protein (CREB). Activation of this pathway modulates gene transcription of numerous cytokines, and results in suppression of TNFα production and eventual inhibition of their proinflammatory and destructive properties [14].

### Pharmacokinetics

The newest and most promising of the PDE4 inhibitors, apremilast, has been evaluated for its pharmacokinetic properties and disposition following oral administration. Multiple daily doses showed rapid absorption (T_max_ = 2 h) and a moderately long half-life (8.2 h) [15].

A separate study monitored healthy male subjects following a single, 20 mg, oral dose and found that apremilast was extensively metabolized via multiple pathways, with unchanged drug representing 45% of the circulating radioactivity and <7% of the excreted radioactivity. Analysis of total radioactivity suggests rapid absorption, with plasma T_max_ values also at 2 h. Mean C_max_ and area under the curve (AUC) values in plasma were 333 ng/ml and 1,970 ng*h/ml, respectively. Metabolic clearance of apremilast was the major route of elimination with the key metabolites demonstrating at least 50-fold less pharmacologic activity than apremilast [16].

Man et al. optimized the structures of a series of 3-(1,3-dioxo-1,3-dihydroisoindol-2-yl)-3-(3,4-dialkoxyphenyl) propionic acid analogues to enhance PDE4 and TNFα inhibitory activity. So far, oral and intravenous administration of these analogues in female rats has shown good pharmacokinetics with low clearance, a moderate volume of distribution, and a 64% oral bioavailability [17].

### Adverse effects

PDE4 is also one of the major phosphodiesterase isoenzymes expressed in the central nervous system, and therefore nausea and emesis are common adverse effects of drug administration [18]. Early PDE4 inhibitors actually failed in clinical trials due to the high prevalence of nausea and emesis [19]. Other adverse effects associated with repeated administration of PDE4 inhibitors include headache, diarrhea, fatigue, dyspepsia, nasopharyngitis, and gastroenteritis [20]. Mesenteric vasculitis is a more worrisome toxicity that may be associated with the PDE4 inhibitors. Studies performed in rodents have demonstrated medial necrosis of the mesenteric arteries after administration of the second generation PDE4 inhibitor cilomilast. However at a meeting convened by the US Food and Drug Administration (FDA) in 2003 to discuss cilomilast in phase III studies, the committee unanimously agreed that the risk of mesenteric vasculitis is not a safety concern based on human studies [21].

The newer PDE4 inhibitor, apremilast, has been well tolerated with few side effects in phase I and II studies. Phase III clinical trials are currently underway and will provide more insight into its dosing and side effect profile. The most frequently reported adverse events have been headache, nausea and pharyngitis (15,34). Researchers used a recognized pharmacophore from the PDE4 inhibitors rolipram and roflumilast in the development of apremilast, and added it to a series of thalidomide analogs in efforts to optimize activity and reduce side effects classically seen with earlier PDE4 inhibitors [22].

### Rolipram

The discovery of the anti-inflammatory actions of PDE4 inhibitors arose from early studies with the prototypic PDE4 inhibitor, rolipram. This was the first selective PDE4 inhibitor investigated, and has been used numerous times for drug comparator studies [23]. Rolipram was also studied as an antidepressant several years before the discovery of its potent PDE4 inhibitory activity [24,25]. Despite its potent anti-inflammatory effects *in vitro*, clinical trials were associated with unacceptably high rates of adverse events, specifically nausea and vomiting [26].

### Roflumilast

Roflumilast was the first, and currently only drug in the PDE4 inhibitor class to be FDA approved. In several countries, this highly selective PDE4 inhibitor is licensed for oral, once-daily treatment of severe chronic obstructive pulmonary disease (COPD). In a pooled analysis of over 6,000 patients receiving roflumilast, higher rates of diarrhea, weight loss, nausea, headache, back pain, insomnia, decreased appetite, and dizziness were reported as compared to those receiving placebo. However the overall rate of adverse events was comparable to that among patients receiving placebo [27].

Pooled results from the pivotal COPD studies M2-124 and M2-125 showed that the weight loss for those in the roflumilast group was generally small (<3% of baseline weight) and typically occurred in the first 6 months of treatment. By the end of the 6 months, almost two-thirds of the weight loss could be attributed to a reduction in fat mass [28]. Prior investigations have revealed a link between PDE4 and lipolysis, possibly through regulation of cAMP pools in human adipocytes and increased plasma glucagon-like peptide 1 (GLP-1) concentrations in rats [29,30].

### Flavonoids

Many flavonoids have been reported to inhibit PDE4 [31,32], and also demonstrate additional anti-inflammatory effects through other pathways. For example, in addition to PDE4 inhibition, the flavonoid dioclein suppresses the production of the inflammatory mediators interleukin (IL)-6, TNFα, a chemokine ligand (CXCL)1/KC, CCL2/JE (monocyte chemotactic protein 1) and nitric oxide (NO), and acts as a scavenger of reactive oxygen species [33]. Studies on dioclein have also demonstrated a synergistic anti-inflammatory effect by targeting multiple pathways [34]. However in contrast to rolipram, dioclein does not selectively inhibit PDE4 with additional activity against PDE1, which may lead to unwanted side effects [35].

### Psoriasis

Psoriasis is a chronic inflammatory autoimmune disorder characterized by inflammatory cell infiltration into the dermis and epidermis accompanied by keratinocyte hyperproliferation [36]. Current treatments, including biological therapies, downregulate cytokine cascades and chemokine production. While these interventions can be highly efficacious, limitations include side effects, intravenous or subcutaneous administration, quality control, and production costs.

AN-2728 is a topically administered, boron-containing compound developed for the treatment of psoriasis. This compound was found to reduce cytokine production, such as TNFα and interferon (IFN)y, and demonstrated activity against the PDE4 enzyme [37]. Several clinical trials of AN-2728 have reported significant effects on markers of efficacy, such as TNFα, in addition to being well tolerated [38].

The determination of cytokines in skin homogenates revealed that both T helper (Th)1 as well as Th2 cytokines are suppressed by PDE4 inhibitors, further proving its utility in the treatment of T cell mediated diseases, such as psoriasis [39].

An open-label, single-arm pilot study investigated the biological and clinical effects of oral apremilast 20 mg once daily in patients with severe plaque-type psoriasis. Of the 19 patients enrolled, 17 completed the study. Of the 19 subjects, 14 (73.7%) showed improvement in their Psoriasis Area and Severity Index (PASI) scores following a 29-day treatment phase. Among these responders, T cells were reduced by 28.8% and 42.6% in the dermis and epidermis, respectively. Epidermal thickness was also reduced by a mean of 20.5% from baseline [15].

More recently, a phase IIb, randomized, multicenter, placebo-controlled, dose-ranging trial for the treatment of plaque-type psoriasis with oral apremilast was completed. Patients were randomly assigned to apremilast 10 mg twice daily, apremilast 20 mg twice daily, apremilast 30 mg twice daily, or placebo. By week 16, a 75% improvement in PASI scores (PASI75) was achieved in 6% (5/88) assigned placebo, 11% (10/89) assigned apremilast 10 mg, 29% (25/87) assigned 20 mg, and 41% (36/88) assigned 30 mg. Significant differences from placebo were seen with apremilast 20 mg and 30 mg (*P* <0.0001), but not 10 mg. Reported adverse events were most frequently mild to moderate and included nausea, upper respiratory tract infection, diarrhea, nasopharyngitis, headache, gastroenteritis, and dyspepsia. Of the eight serious adverse events, none were judged to be related to apremilast [40].

Currently there are two phase III, double-blind, placebo-controlled, multicenter trials (ESTEEM 1 (NCT01194219) and ESTEEM2 (NCT01232283)) investigating the use of oral apremilast 30 mg in adults with moderate to severe plaque psoriasis. These trials include a 52 week randomized, blinded, placebo-controlled phase, with primary endpoints measured at week 16, in addition to a 4-year extension phase [40].

### Psoriatic arthritis

A phase II, multicenter, randomized, double-blind, placebo-controlled study enrolled 168 subjects with psoriatic arthritis (PsA) during a 12-week treatment phase. Subjects were randomized to 20 mg apremilast twice daily, apremilast 40 mg once daily, or placebo. After completing the initial 12-week phase, subjects receiving placebo were given a 12-week course of apremilast. Following the treatment phase in both groups, subjects participated in a 4-week observation phase. The primary endpoint was the proportion of subjects achieving the American College of Rheumatology criteria for 20% improvement (ACR20) at week 12.

In total, 44% of actively treated patients achieved the primary endpoint of ACR20 compared with 12% of the placebo cohort (*P* <0.001). The study revealed promising results for the treatment of PsA with oral apremilast, but was limited by the relatively short duration and unclear long-term efficacy and safety data. Additionally, the 90% of subjects enrolled were white and therefore the study may lack generalizability. Lastly, prior systemic therapy for PsA may alter the efficacy of apremilast and was not examined in this study.

The most common adverse events (AEs) were diarrhea, nausea, headache, fatigue, and nasopharyngitis with 84.3% of subjects in the treatment phase reporting at least one AE. However most events were mild to moderate and no clinically relevant laboratory or electrocardiographic abnormalities were reported [41].

Results of this study are encouraging, and phase III clinical trials are currently underway. The efficacy and tolerability of apremilast in patients with psoriatic arthritis are now being studied in four independent phase III studies (PALACE 1 (NCT01172938), PALACE 2 (NCT01212757), PALACE 3 (NCT01212770), and PALACE 4 (NCT01307423)) [40]. These studies include both patients who have received disease-modifying antirheumatic drugs and those who have not.

### Ankylosing spondylitis

Manifestations of ankylosing spondylitis (AS) include axial and peripheral skeletal inflammation, fat infiltration, and new bone formation. Therapeutic response centers on patient-reported outcomes such as pain, mobility and function as well as objective measures such as inflammation, and new bone formation that can be visualized by magnetic resonance imaging (MRI) and conventional radiography [42-44]. Moreover, the degree of clinical response with treatment may also correlate with fluctuations in biomarkers [45-47].

Recently, updated management guidelines published by the Assessment of SpondyloArthritis (ASAS) and the European League Against Rheumatism (EULAR) report that there is no evidence for the efficacy of disease-modifying antirheumatic drugs (DMARDs) such as methotrexate and sulfasalazine for the treatment of axial disease, leaving patients with limited treatment options. The two classes of drugs that have been shown to reduce the signs and symptoms of AS include non-steroidal anti-inflammatory drugs (NSAIDs) and TNFα blockers [48,49].

Nevertheless, at the 2011 ACR meeting, results from a small pilot study were presented showing that apremilast may be efficacious in patients with longstanding AS. This double-blind, placebo controlled phase II unpowered pilot study included 36 subjects with longstanding AS who had not sufficiently responded to NSAIDs over 12 weeks. Of these subjects, 17 received apremilast 30 mg twice daily compared to 19 who received placebo. The apremilast group also saw a significant change from baseline (mean percentage) in levels of receptor activator of nuclear factor κB (NFκB) ligand (RANKL) and sclerostin [50].

### Rheumatoid arthritis

Rheumatoid arthritis (RA) is another chronic, inflammatory autoimmune disease and primarily targets the synovial tissues of joints. Local production of cytokines and chemokines leads to leukocyte infiltration, and eventual erosion of cartilage and bone [51,52]. TNFα has been shown to promote cytokine and chemokine production, as well as cellular activation and articular destruction in RA [53].

Given the pathophysiologic characteristics of RA, a study was performed to assess the anti-inflammatory effects of apremilast in human synovial cells collected from rheumatoid arthritis patients, as well as two murine models of arthritis. These synovial cells were cultured in the presence of increasing concentrations of apremilast for 48 h and enzyme-linked immunosorbent assay (ELISA) was used to analyze spontaneous TNFα production.

Results from this study showed that apremilast led to a dose-dependent inhibition of spontaneous production of TNFα from human rheumatoid synovial membrane cultures. Additionally, both murine models showed a significant reduction in arthritis clinical scores over a ten-day treatment period with apremilast. Healthy joint architecture was also maintained in a dose-dependent manner. Unlike the first generation PDE4 inhibitor rolipram, apremilast did not demonstrate any adverse effects in treatment-naïve mice, possibly due to enhanced selectivity of apremilast [54].

An interim analysis of the data from a phase II pilot study investigating the use of apremilast in combination with methotrexate reported that the primary endpoint of ACR20 was not reached [55]. A placebo-controlled phase II trial using apremilast as monotherapy for RA is currently underway [56].

### Systemic lupus erythematosus

Systemic lupus erythematosus (SLE) is a chronic autoimmune disease that can affect a variety of organs and is predominantly seen in women. Treatment is focused on controlling symptoms and often involves the use of corticosteroids and other systemic immunosuppressant therapies [57,58].

A recent study targeted increased PDE4 activity in lupus conditions using MRL/lpr mice (a mouse model developing severe lupus disease). Four groups of female MRL/lpr mice were injected at 5, 7, 9 and 13 weeks with one of ethanol, pentoxifylline, denbufylline, or NCS 613 (a novel PDE4 inhibitor). Results showed that both the survival time and the appearance of proteinuria of NCS 613-treated mice are significantly delayed, both with *P* values of 0.005 [59]. While study size was limited, the results demonstrate potential for the use of PDE4 inhibitors in patients with SLE.

### Sarcoidosis

Previous reports have described the use of PDE4 inhibitors in patients with the systemic inflammatory disease, sarcoidosis. While pentoxifylline has been shown to be effective, use of this drug is limited by associated adverse events [60].

A small study evaluated the use of apremilast in 15 subjects who had failed systemic therapies for sarcoidosis. Patients received oral apremilast 20 mg twice daily, with a reduction to once daily dosing following the onset of any adverse event. Only two patients needed dosage reduction due to jitteriness in one patient and nausea in the other. No further adverse events were reported following this change in dose.

Active lesions were assessed during 12 weeks of therapy with the Sarcoidosis Activity and Severity Index (SASI) as well as with photographs taken at baseline and at week 12. Photographs were presented in random order to three assessors and were scored from 1 to 5 (1 much better, 5 much worse).

Results showed a significant reduction in SASI induration scores at weeks 4 and 12. The normalized mean score given by the assessors after 12 weeks of therapy was 2/5 (somewhat better after therapy), with good inter-reader consistency. Of note, three patients developed significant worsening of their cutaneous lesions within 3 months after discontinuation of apremilast [61].

### Inflammatory bowel disease

The group of inflammatory conditions affecting the colon and small bowel known as inflammatory bowel disease can present in patients with life-altering symptoms lasting weeks to months at a time. Both Crohn’s disease and ulcerative colitis (UC) present with diarrhea, bleeding, fecal urgency and incontinence, abdominal pain, and fever caused by inflammation in the gut.

The long-term therapeutic goal for these patients focuses on inducing and maintaining remission of symptoms to improve patient quality of life [62]. Existing anti-inflammatory agents such as 5-aminosalicyclates and other immunosuppressants have limitations due to adverse drug reactions, loss of therapeutic response, or lack of response in some patients [63].

Like many autoimmune diseases, the inflammation in inflammatory bowel disease (IBD) has been linked to upregulation of proinflammatory cytokines, such as TNFα, and nuclear translocation of the proinflammatory transcription factor complex NFκB [64]. TNFα is thought to damage the gut via upregulation of matrix metalloproteinase (MMP) production by gut myofibroblasts, leading to breakdown of extracellular matrix, tissue damage, and ulcer formation [65]. The quantity of NFκB activation has also been shown to correlate with the degree of mucosal inflammation and disease activity, as well as TNFα upregulation. NFκB activation works in a positive feedback to induce TNFα, perpetuating further inflammation and disease processes [66].

Given the suppression of TNFα and NFκB associated with a variety of PDE4 inhibitors, two phase III clinical trials (FACT I and FACT II) were designed. These studies investigated the safety and efficacy of the PDE4 inhibitor, tetomilast, in treating moderately severe UC. Both studies were multicenter, randomized, double-blind, placebo-controlled, parallel-arm, dose comparison studies of tetomilast in subjects with active UC.

Tetomilast was not found to have a significant effect on individual symptom or sigmoidoscopy scores. However, there was a trend for tetomilast to improve severity of rectal bleeding from baseline when compared to placebo (*P* = 0.017). By week 8, the efficacy scores had improved for those patients receiving tetomilast (with or without concomitant 5-aminosalicylic acid) compared to those on placebo, although these results were not significant statistically. One potential reason for the lack of difference in the therapeutic response may be the very high placebo response rates seen in patients with IBD. Both 25 and 50 mg of tetomilast were generally well tolerated in subjects with active UC with no major adverse events [63].

### Atopic dermatitis

Atopic dermatitis is a chronic inflammatory disease characterized by eczematous lesions and intense itching. Inflammatory infiltrates in these cutaneous lesions consist of T lymphocytes, neutrophils, eosinophils, monocytes, macrophages, and mast cells [67]. High levels of PDE4 activity are also found in leukocytes of these patients [68].

A study in Japan looked at the effects of PDE4 inhibitors, cilomilast, roflumilast, and rolipram on induced dermatitis in mice models. Cilomilast, roflumilast, and to a lesser extent rolipram, suppressed myeloperoxidase (MPO) activity, a quantitative index of neutrophils accumulating in skin associated with chronic inflammation. After 18 days of treatment, cilomilast and roflumilast showed a 47 and 36% recovery in skin severity score, respectively. This effect was more potent than the 25% recovery seen with cyclosporine A, especially in the earlier stages of treatment [69].

A subsequent study by Harada et al. used the PDE4B inhibitor, KF66490 to treat induced AD in mouse models. KF66490 significantly inhibited increases in ear thickness, IL-4 and IL-1B levels, and proliferation of fibroblasts and CD3-positive T cells. Compared to the first generation PDE4 inhibitor, rolipram, KF66490 also produced less potent emetic effects [70].

More recently, an open-label prospective trial of apremilast in 16 patients with moderate to severe AD was conducted to assess the safety, efficacy, and possible mechanism of action of apremilast in AD. One cohort consisted of six subjects treated with apremilast 20 mg twice daily for 3 months, while the second cohort consisted of ten subjects treated with apremilast 30 mg twice daily for 6 months. Participants in the study were required to remain on triamcinolone acetonide 0.1% for 2 weeks prior to the start of the study as well as throughout the trial. Nausea, the most common adverse event, was rated as mild and improved over the course of the study in all patients. After 3 months of treatment, a significant reduction of itch from baseline (VAS) and improvement in quality of life (assessed via Dermatology Life Quality Index (DLQI) score) was seen in cohort 1 (*P* = 0.02 and *P* = 0.003, respectively), while Eczema Area and Severity Index (EASI) and quality of life (DLQI) scores improved in cohort 2 (*P* = 0.008 and *P* = 0.01, respectively). At 6 months, statistically significant improvement was seen in all outcomes in cohort 2, including VAS (*P* = 0.03), DLQI (*P* = 0.03), and EASI (*P* = 0.002) [71].

### Alzheimer’s disease

PDE4 inhibitors have also been investigated in the treatment of patients with Alzheimer’s disease. The neuropathological changes seen in Alzheimer’s are closely linked to chronic inflammation and apoptosis, with elevations of biomarkers seen even in early stages of disease [72].

Accumulation of amyloid beta (Aβ) peptides has been shown to produce inflammatory responses [73], activate the apoptotic pathway [74], inhibit hippocampal synaptic plasticity, and impair memory [75]. Similar responses were induced by infusion of aged Ab25-35 into the hippocampus, with reversal of memory deficits seen following repeated treatment with rolipram. These positive findings may be at least partially attributed to the blockade of inflammatory responses and apoptosis mediated by cAMP/CREB signaling. In fact, increased levels of pCREB were reported in the hippocampus following treatment with rolipram. These results may suggest a potential role for the use of PDE4 inhibitors in treatment of memory loss in patients with Alzheimer’s [75].

### Multiple sclerosis

The demyelinating autoimmune disease, multiple sclerosis (MS), has often been studied in animal models by inducing experimental autoimmune encephalomyelitis (EAE) in genetically susceptible animals [76]. The EAE model also mimics the relapsing-remitting presentation of MS seen in humans.

One study demonstrated a reduction in the clinical signs of EAE in mice models during the administration of rolipram. Improvement was seen during both the initial presentation of disease as well as subsequent relapses [77].

Matrix metalloproteinases (MMPs) are a gene family of zinc-dependent endopeptidases involved in the proteolytic modeling of the extracellular matrix as well as the pathogenesis of several autoimmune disorders of the peripheral and central nervous systems, such as MS [78]. *In vitro* models have shown that rolipram inhibits NFκB, a key regulator of inflammatory processes and gene expression related to EAE and MS, including MMP-9 [79].

When rats primed to EAE were treated with rolipram, the high levels of NFκB activation in freshly obtained cells were prevented. Furthermore, inhibition was also seen after incubation of encephalitogenic cells with rolipram, indicating that interference with NFκB activation is a direct effect of the drug. Inhibition of NFκB was also accompanied by a decrease in MMP-9 gene expression [80].

## Conclusions

Advancements in phosphodiesterase-targeted therapies have shown promise in recent years for treating patients with a variety of autoimmune diseases. After the initial investigations of the prototypic PDE inhibitor rolipram, more selective inhibitors targeting the PDE4 isozyme have been developed. With phase II and phase III clinical trials currently underway to evaluate the safety and efficacy of the latest generation of PDE4 inhibitors, namely apremilast, a new class of treatments may be around the corner for patients suffering from chronic, autoimmune diseases.

## Abbreviations

Aβ: Amyloid beta; ACR20: American college of rheumatology criteria for 20% improvement; AE: Adverse event; AS: Ankylosing spondylitis; ASAS: Assessment of spondyloarthritis; cAMP: Cyclic adenosine monophosphate; CREB: cAMP-response element binding protein; COPD: Chronic obstructive pulmonary disease; CXCL: Chemokine C-X-C motif ligand; DLQI: Dermatology life quality index; DMARD: Disease-modifying antirheumatic drug; EAE: Experimental autoimmune encephalomyelitis; EASI: Eczema area and severity index; ELISA: Enzyme-linked immunosorbent assay; EULAR: European League Against Rheumatism; GLP-1: Glucagon-like peptide; IFN: Interferon; IL: Interleukin; MMP: Matrix metalloproteinase; MPO: Myeloperoxidase; MS: Multiple sclerosis; NFκB: Nuclear factor κB; NO: Nitric oxide; NSAID: Non-steroidal anti-inflammatory drug; PASI: Psoriasis area and severity index; PDE: Phosphodiesterase; PKA: Protein kinase A; PsA: Psoriatic arthritis; RA: Rheumatoid arthritis; RANKL: Receptor activator of NFκB ligand; SASI: Sarcoidosis activity and severity index; SLE: Systemic lupus erythematosus; TNF: Tumor necrosis factor; UC: Ulcerative colitis; VAS: Visual analog scale.

## Competing interests

ABG is on the advisory boards of Abbott Laboratories, Actelion, Amgen, Astellas Pharma US Inc., Belersdorf Inc., BMS, Celgene Corporation, Coronado Biosciences, Janssen-Ortho Inc., Novo Nordisk A/S, Pfizer Inc., and UCB. ABG is a consultant to Abbott Laboratories, Amgen, Canfite, Celgene Corporation, Incyte Corporation, Lilly ICOS LLC, Merck & Co. Inc., Novartis Pharmaceuticals Corp., and Teva. ABG holds an investigator role for Abbott Laboratories, Celgene Corporation, Immune Control, Janssen-Ortho Inc., Lilly ICOS LLC, Novartis Pharmaceuticals Corp., Novo Nordisk A/S, Pfizer Inc., and UCB. ABG has received an institutional grant from Amgen, and honoraria from DermiPsor. NKu, AMG and NKi have no competing interests.

## Authors’ contributions

All authors made substantial contributions to the drafting, reading and editing, and approved the final manuscript.

## Pre-publication history

The pre-publication history for this paper can be accessed here:

http://www.biomedcentral.com/1741-7015/11/96/prepub
